# Prediction of Biochemical Recurrence After Radical Prostatectomy Based on Preoperative ^68^Ga-PSMA-11 PET/CT

**DOI:** 10.3389/fonc.2021.745530

**Published:** 2021-09-30

**Authors:** Xuefeng Qiu, Mengxia Chen, Haoli Yin, Qing Zhang, Haoyang Li, Suhan Guo, Yao Fu, Shiming Zang, Shuyue Ai, Feng Wang, Hongqian Guo

**Affiliations:** ^1^ Department of Urology, Affiliated Drum Tower Hospital, Medical School of Nanjing University, Nanjing, China; ^2^ Institute of Urology, Nanjing University, Nanjing, China; ^3^ Department of Radiology, Affiliated Drum Tower Hospital, Medical School of Nanjing University, Nanjing, China; ^4^ Department of Applied Mathematics, X2017 École Polytechnique, Palaiseau, France; ^5^ School of Artificial Intelligence, Nanjing University, Nanjing, China; ^6^ Department of Pathology, Affiliated Drum Tower hospital, Medical School of Nanjing University, Nanjing, China; ^7^ Department of Nuclear Medicine, Nanjing First Hospital, Nanjing Medical University, Nanjing, China

**Keywords:** prostate cancer, PSMA - prostate specific membrane antigen, radical prostatectomy, biochemical recurrence (BCR), prediction

## Abstract

**Purpose:**

This study was designed to investigate the prognostic role of preoperative ^68^Ga-PSMA-11 PET/CT in predicting biochemical recurrence (BCR) of localized prostate cancer (PCa) after radical prostatectomy (RP).

**Methods:**

A total of 77 biopsy-confirmed PCa patients with ^68^Ga-PSMA-11 PET/CT prior to RP were included. A PSMA-ligand PET/CT-based risk model with SUV_max_, maximum diameter of the index tumor and T stage was developed for prediction of 2-year BCR using Cox regression analysis. Also, the efficacy of the developed risk model was compared with European Association of Urology risk stratification (D’Amico) and the Cancer of the Prostate Risk Assessment (CAPRA) score. C-index and calibration plot were used to assess discrimination and calibration with internal validation.

**Results:**

With a median follow-up of 25 months, 23 (29.9%) patients experienced BCR within 2 years after RP. Patients experienced BCR had a significant higher PSA at diagnosis (p<0.001), a higher ISUP grade of biopsy (p=0.044), as well as a higher ISUP grade (p=0.001), a higher possibility of T3 diseases (p=0.001) and positive margin (p=0.008) on postoperative pathology. SUV_max_, maximum diameter of the index tumor and T stage on preoperative PSMA-ligand PET/CT were significantly associated with BCR (all p<0.01). PSMA-ligand PET/CT-based risk model had a superior discrimination (c-index 78.5%) and good calibration at internal validation. The efficacy of this model in predicting 2-year BCR after RP was better, compared with CAPRA (c-index 66.3%) and D’Amico (c-index 66.2%). The addition of the PSMA-ligand PET/CT-derived variables also improved the efficacy of the existing models in predicting 2-year BCR (C-index of 78.9% for modified CAPRA and 79.3% for modified D’Amico, respectively).

**Conclusion:**

A PSMA-ligand PET/CT-based risk model showed good efficacy in predicting 2-year BCR after RP, which needed to be validated by further prospective studies.

## Introduction

Radical prostatectomy (RP) is a widely adopted definitive option for men with localized prostate cancer (PCa) (1, 2). However, up to 40% of patients experienced biochemical recurrence (BCR) after RP (3). Several clinical models, such as D’Amico risk stratification scheme (4), and the University of California, San Francisco, Cancer of the Prostate Risk Assessment (CAPRA) score, have been developed to predict BCR (5). Preoperative variables such as prostate-specific antigen (PSA), clinical T staging, and Gleason score of systematic biopsy are used as prognostic factors in these models. However, the efficacy of these nomograms are far from excellent, with the prediction accuracy of 5-year BCR less than 70% (6, 7).

Prostate specific membrane antigen (PSMA)-ligand positron emission tomography/computed tomography (PET/CT) is currently a promising technique for recurrent PCa imaging (8, 9), as well as primary staging (10, 11). Our previous study indicates improved sensitivity of PSMA-ligand PET/CT in describing intraprostatic tumor lesions compared with multiparametric magnetic resonance Imaging (mpMRI) (12). In addition, increased PSMA uptake on PSMA-ligand PET/CT has been indicated to be positively correlates with prostate cancer aggressiveness and adverse pathologic features in our previous studies (13, 14), making PSMA-ligand PET/CT a potential tool to predict BCR following RP. Nonetheless, current models for prediction of BCR are mostly based on clinical and pathologic variables. The predictive role of PSMA-ligand PET/CT in this setting has been rarely investigated (15). Furthermore, the added value of PSMA-ligand PET/CT over the pre-existing models has not been evaluated.

Therefore, this study was designed to assess the potential role of PSMA-ligand PET/CT as a biomarker to predict early BCR after RP. We developed a PSMA-ligand PET/CT-based risk model for the prediction of BCR. The added value of PSMA-ligand PET/CT to the commonly used clinical models to predict BCR was also evaluated.

## Patients and Methods

### Study Population

We retrospectively included 138 consecutive patients with biopsy-confirmed prostate cancer who underwent ^68^Ga-PSMA-11 PET/CT before radical prostatectomy (RP) between January 2017 and June 2019. We excluded the patients with suspicious pelvic lymph nodes (n=11) or distant metastases (n=5). Patients who received treatment before RP (TURP, n=2; hormone therapy, n=35) were also excluded. Patients with inadequate clinical or pathological information (n=3) or incomplete follow-up information were also excluded (n=5). Finally, 77 patients were eligible for the analysis. This study was approved by the Ethics Committee of the Drum Tower Hospital (2017-147-01).

### PSMA-Ligand PET/CT Scanning and Image Evaluation

^68^Ga-PSMA-11 PET/CT was acquired as previously described (12). ^68^Ga-PSMA-11 was synthesized using an ITG semiautomated module and were injected intravenously one hour before scanning. All PET/CT scans were performed in an uMI 780 PET/CT scanner (United Imaging Healthcare (UIH), Shanghai, China). A CT scan (130 keV, 80 mAs) and a static emission scans, corrected for dead time, scatter and decay, were acquired from the vertex to the proximal legs. PSMA-ligand PET/CT imaging were double reviewed by two experienced nuclear medicine physicians (SZ and SA). Lesions were delineate by higher uptake than background or blood pool. Semi-quantitative analysis of PSMA intensity was evaluated by an automated standard maximum uptake value (SUV_max_) in the delineated lesion. For patients with multiple lesions, the one with highest SUV_max_ was recognized as the index tumor. The maximum diameter of the index tumor was also measured based on the delineate lesions previous recognized by nuclear medicine physicians on PET imaging as primary tumor is not distinctly visible on CT alone. For the assessment of T stage on PSMA-ligand PET/CT, all the assessment were based on the fusion image of PET and CT. PET image with angulated contour of the prostate gland or obliteration of the recto-prostatic angle accordant with the shape on CT were recognized as extracapsular extension (T3a) while seminal vesicle invasion (T3b) was diagnosed if there is a focal or diffuse ^68^Ga-PSMA-11 accumulation above the background (16).

### Covariates, Endpoints, and Model Development

Clinical information including age, PSA level at diagnosis and clinical stage assessed by digital rectal examination (DRE) were included. Transperineal systematic prostate biopsy were performed, with additional fusion targeted biopsies if suspicious lesions (PI-RADS 3-5) were detected on multiparametric magnetic resonance imaging (mpMRI). For preoperative parameters of biopsy, Gleason score and percentage of positive cores were collected. For, PSMA-ligand PET/CT-derived parameters, we included SUV_max_, maximum diameter of the index tumor, and T stage. Postoperative BCR was defined as three successive rises in PSA level of >0.1 ng/ml at least 6 weeks postoperatively with final PSA >0.2 ng/ml (n=19), or administration of secondary therapy for evidence of detectable PSA >0.1 ng/ml at least 6 weeks postoperatively (n=10) (17).

PSMA-ligand PET/CT-based model was developed by inputting PSMA-ligand PET/CT-derived variables (SUV_max_, maximum diameter of the index tumor, and T stage). For the existing clinical models, D’Amico and CAPRA scores were collected according to the established D’Amico and CAPRA risk stratification scheme (5), by inputting clinical variables such as patient age, PSA level at diagnosis, Gleason score at biopsy, percentage of positive cores at biopsy, and clinical T stage assessed by DRE. To investigate the added value of PSMA-ligand PET/CT-derived parameters to the existing clinical models, modified D’Amico and modified CAPRA were developed. D’Amico score or CAPRA score was integrated with SUV_max_, maximum diameter and T stage and re-assessed by Cox regression analyses.

### Statistical Analysis

Mann-Whitney U test was performed for continuous variables and the Fisher exact test/chi-square test for categorical variables to compare the characteristics between the patients who underwent BCR and those free from BCR at 2-year follow-up. The risk of BCR was predicted using Cox regression model. By plotting the observed versus predicted cumulative incidences within 2 years after RP, we also assessed the calibration of our risk model. The discrimination of our risk model and modified D’Amico or CAPRA models was assessed by the concordance index (C-Index). The C-index and calibration plots were produced using the predicted probabilities after a validation with bootstrap by 1000 iterations. A significance level of 5% was used. All analyses were performed using SPSS software, version 22.0 (IBM Corp.) and R statistical package v.3.0.2 (R Project for Statistical Computing, www.r-project.org).

## Results

### Patient Characteristics and Survival Analysis

**Table 1** showed the clinical, preoperative and postoperative pathological characteristics as well as the PSMA-ligand PET/CT-derived features of the 77 patients, with a median age of 69 (interquartile range [IQR]: 62–73 years and median PSA 13.30 ng/ml (IQR: 7.89-28.70) at diagnosis. The median (IQR) follow-up time were 25 (19-27) months for all patients, 25 (21.5-26.8) months for BCR patients and 26 (17.5-27) months for BCR-free patients. Twenty-nine (37.7%) and 23 (29.9%) patients experienced BCR overall and within 24 months after RP. The patients were divided into two groups according to the status of BCR at 2-year follow-up. All clinical, pathological, and imaging variables were compared between the two groups.

**Table 1:** Characteristics of prostate cancer patients with ^68^Ga-PSMA PET/CT scanning prior to radical prostatectomy.

Characteristics	Total (n = 77)	Median (IQR) or n (%)		p
		BCR Free (n = 54)	BCR (n = 23)	
**Preoperative characteristics**				
Age	69 (65-75)	69 (65-74)	68 (65.5-75.5)	0.993
PSA	13.30 (7.89-28.70)	10.89 (6.61-16.00)	32.25 (14.05-71.43)	**0.000**
Clinical T stage by DRE				0.356
T2	71 (92.2)	51 (94.4)	20 (87.0)	
T3	6 (7.8)	3 (5.6)	3 (13.0)	
ISUP at Biopsy				**0.044**
1	14 (18.2)	13 (24.1)	1 (4.3)	
2	18 (23.4)	13 (24.1)	5 (21.7)	
3	16 (20.8)	13 (24.1)	3 (13.0)	
4	21 (27.3)	10 (18.5)	11 (47.8)	
5	8 (10.4)	5 (9.3)	3 (13.0)	
Percent of positive cores on biopsy	35.71 (21.42-55.91)	30.0 (21.4-51.6)	42.9 (28.1-57.64)	0.130
**Postoperative characteristics**				
Post-operative ISUP				**0.001**
1	5 (6.5)	5 (9.3)	0 (0)	
2	25 (32.5)	22 (40.7)	3 (13.0)	
3	18 (23.4)	15 (27.8)	3 (13.0)	
4	16 (20.8)	7 (13.0)	9 (39.1)	
5	13 (16.9)	5 (9.3)	8 (34.8)	
Pathological T stage, n (%)				**0.001**
T2	27 (35.5)	23 (43.4)	4 (17.4)	
T3a	35 (46.1)	26 (49.1)	9 (39.1)	
T3b	14 (18.4)	4 (7.5)	10 (43.5)	
Positive margin				**0.008**
Absent	56 (72.7)	12 (52.2)	44 (81.5)	
Present	21 (27.3)	11 (47.8)	10 (18.5)	
**Preoperative PET/CT features**				
SUV_max_	13.04 (7.76-21.60)	10.70 (6.83-17.00)	22.90 (15.74-31.01)	**0.000**
Maximum diameter (cm)	1.19 (0.76-2.27)	1.09 (0.74-1.80)	1.93 (1.13-2.44)	**0.008**
PET-detected T stage				**0.002**
T2	46 (59.7)	39 (72.2)	7 (30.4)	
T3a	23 (29.9)	12 (22.2)	11 (47.8)	
T3b	8 (10.4)	3 (5.6)	5 (21.7)	

PSMA, prostate specific membrane antigen; PET/CT, positron emission computed tomography; IQR, interquartile range; BCR, biochemical recurrence; PSA, prostate specific antigen; DRE, digital rectal examination; SUV, standard uptake value; ISUP, International Society of Urological Pathology; cm, centimeter.

Significant P values were presented in bold text.

At the time point of 24-month follow-up, 23 (29.9%) had experienced BCR while the other 54 (70.1%) are free from BCR. **Table 1** showed summary characteristics of the two groups. The BCR group had a significantly higher PSA level at diagnosis (32.25 versus 10.89 ng/ml), a higher ISUP grade of biopsy (p=0.044), as well as a higher ISUP grade (p=0.001), a higher possibility of T3 diseases (p=0.001) and positive margin (p=0.008) on postoperative pathology. For parameters on PSMA-ligand PET/CT, patients with BCR had a higher SUV_max_, a larger maximum diameters and a higher T stage than BCR-free patients.

### Multivariable Models Predicting BCR

**Figure 1:**
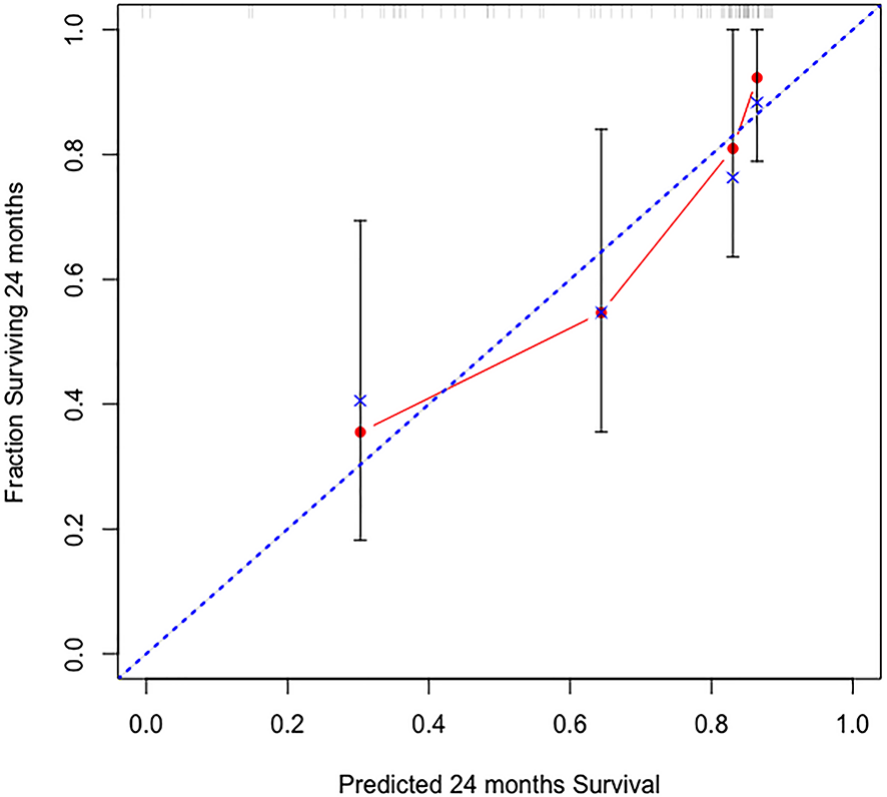
Calibration plot of observed proportion versus predicted probability of 2-year biochemical recurrence after radical prostatectomy by the PSMA-ligand PET/CT-based risk model.

In Cox regression, PSMA-ligand PET/CT-based model with input of SUV_max_, maximum diameters, and T stage on PSMA-ligand PET/CT achieved a superior discrimination of BCR during the 2-year follow-up than CAPRA [C-Index: 78.5% (70.3-86.7%) *versus* 66.3 (76.5-56.1)] and D’Amico [C-Index: 78.5% (70.3-86.7%) *versus* 66.2 (75.0-57.4)] (**Table 2**). This model was also characterized by a good calibration at internal validation (**Figure 1**). The inclusion of the PSMA-ligand PET/CT-derived variables also improved the efficacy of the existing models in predicting post-surgery BCR (C-Index: 66.3 versus 78.9% for CAPRA and modified CAPRA; C-Index: 66.2 versus 79.3 for D’Amico and modified D’Amico) (**Table 2**).

**Table 2:** Cox regression analyses assessing the prediction models of biochemical recurrence in prostate cancer patients treated with radical prostatectomy.

Parameters	PET/CT based risk model		D’Amico		CAPRA		Modified D’Amico		Modified CAPRA	
	HR (95% CI)	p	HR (95% CI)	p	HR (95% CI)	p	HR (95% CI)	p	HR (95% CI)	p
PET/CT T stage										
T2	1 (ref)		**-**	**-**	**-**	**-**	1 (ref)		1 (ref)	
T3a	2.92 (1.08-7.87)	**.034**	**-**	**-**	**-**	**-**	2.64 (0.38-7.15)	.055	2.76 (1.02-7.47)	.045
T3b	2.29 (0.54-9.72)	.260	**-**	**-**	**-**	**-**	1.74 (0.57-7.29)	.449	1.99 (0.50-8.61)	.358
SUV_max_	1.04 (1.02-1.07)	**.002**	**-**	**-**	**-**	**-**	1.04 (0.96-1.07)	**.003**	1.04 (1.01-1.07)	**.004**
Maximum diameter on PET	0.97 (0.58-1.61)	.905	**-**	**-**	**-**	**-**	0.88 (1.14-1.51)	.632	0.90 (0.59-1.56)	.686
D’Amico score	**-**	**-**	2.71 (1.37-5.38)	**0.004**	**-**	**-**	2.01 (0.50-4.41)	.083	**-**	**-**
CAPRA score	**-**	**-**	**-**	**-**	1.30 (1.09-1.56)	**0.004**	**-**	**-**	1.11 (0.90-1.38)	.363
C-Index	78.5 (70.3-86.7)		66.2 (57.4-75.0)		66.3 (56.1-76.5)		79.3 (70.1-88.5)		78.9 (70.4-87.3)	

PET/CT, positron emission computed tomography; HR, hazard ratio; CI, confidence intervals; SUV, standard uptake value.

Significant p values were presented in bold text.

## Discussion

The newly developed risk model based on ^68^Ga-PSMA-11 PET/CT-derived parameters (SUV_max_, maximal diameter of index tumor, and T staging) showed better performance in predicting 2-year BCR, compared with that of D’Amico and CAPRA models. Furthermore, we found that addition of parameters obtained from PSMA-ligand PET/CT outperformed models based on clinical and biopsy variables. To the best of our knowledge, this was the first study to develop a PSMA-ligand PET/CT-based model for prediction of BCR after RP.

The evidence investigating the role of PSMA-ligand PET/CT in predicting BCR after RP was very limited. Roberts et al. showed that intraprostatic ^68^Ga-PSMA-11 intensity (SUV_max_) was one of the significant pre-operative predictors of progression-free survival after RP. Sub-analysis indicated that SUV_max_ was the most significant predictor of progression-free survival in patients with biopsy Gleason score ≤ 4 + 3 (15). Our study developed a new risk model only based on parameters derived from PSMA-ligand PET/CT, which was different from Roberts’s study that SUV_max_ was added to clinical and pathological variables for Cox regression analysis. In addition, the comparison of the efficacy to the existing clinical models was not performed in Roberts’s study.

In the present study, our risk model showed better performance compared to the commonly used D’Amico and CAPRA models (**Table 2**), although only three parameters (SUV_max_, T staging, and tumor size described on PSMA PET/CT) were included. This result might be explained by the better performance of PSMA-ligand PET/CT-derived parameters in indicating histopathological features compared with clinical parameters. The most commonly used clinical variable reflecting tumor aggressiveness was PSA. However, PSA was an organ-specific biomarker instead of a disease-specific biomarker (18), as it could be induced to be released by several benign diseases such as benign prostatic hyperplasia (BPH), and prostatitis (19). In contrast, PSMA could be considered as PCa-specific marker, as it was highly expressed on the surface of PCa cells (20, 21). The other clinical variable that reflected tumor aggressiveness was histopathology obtained from prostate biopsy. However, Gleason score of prostate biopsy was always related with underestimation of tumor aggressiveness, as Gleason score upgrading from systematic biopsy to RP was commonly reported (22). Though MRI-targeted biopsy increased the detection rate of clinical significant PCa, it was associated with a 30.9% upgrading of cancer group (23). It might be due to the relatively low sensitivity of mpMRI in detecting intraprostatic lesions, especially for small lesions with low grade (24). Moreover, only small part of tissue was obtained from targeted biopsy, which was difficult to reflect the tumor grade of the whole lesion. It has been demonstrated that the detection rate was improved when the number of targeted biopsy increased (25). Different from biopsy, preoperative PSMA-ligand PET/CT was more informative for tumor grade reporting. Previous studies had revealed that SUV derived from PSMA PET/CT was positively correlated with tumor Gleason score (26, 27).

T staging on PSMA-ligand PET/CT is another contributor for better performance of our risk model in predicting of BCR compared with clinical models (**Table 2**). Clinically, tumor staging is assessed by DRE. Apparently, PSMA-ligand PET/CT provides more precise information regarding tumor size and tumor location compared with DRE, improving the efficacy of PSMA-ligand PET/CT in evaluating clinical staging of T1 and T2. Recently, accumulative evidence shows the equivalent and even improved efficacy of PSMA-ligand PET/CT in detecting extraprostatic extension (EPE) and seminal vesical invasion (SVI) compared with mpMRI (16, 28), which could explain the significantly improved efficacy of PSMA-ligand PET/CT in providing tumor information regarding T3 staging. As shown in **Table 2**, T3a on PSMA-ligand PET/CT was significantly associated with the higher BCR after RP. However, this was not observed in patients with T3b, which has been reported to be a strong risk factor for BCR. It might be due to the smaller sample size in this polit study. Only 8 patients (10.4%) (**Table 1**) with T3b staging on PSMA-ligand PET/CT were included in the present study.

In our study, about 30% (23/77) patients experienced BCR within 2-year follow-up post PR, which was higher than the published results (29). Since tumor grade had been well demonstrated to be an independent predictor for early BCR after RP (5), our results could be explained by more cases with higher Gleason score (preoperative ISUP>2: 58.5% versus 36%) (30). Also, the median PSA level in the present study was much higher compared with that in the published study (13.30 versus 7.49 ng/ml) (30). Different from the United States, PCa screening was less pervasive in China, resulting in much higher percentage of high/very high risk and even metastatic patients at initial diagnosis (31). Therefore, the efficacy of our risk model for low-to-intermediate risk cases needed to be further validated with external data.

Regarding limitations, our study was a single-center retrospective study with relatively small sample and the median follow-up was only 25 months for patients without BCR. Therefore, our model needed to be further validated on patients with a longer follow-up procedure, as they might experience BCR after maintaining BCR-free survival within this period. To avoid selection bias, our risk model needed to be validated by further prospective studies before clinical application, as patients with pelvic lymph nodes and distant metastases were not included. However, our study aimed to propose the perspective that PSMA-ligand-based risk model might have great potential for risk stratification and prediction of BCR after RP, as it could provide noninvasive and prospective information regarding tumor aggressiveness and prognosis. In our established model, the weight of maximal tumor diameters seemed to be limited compared with T stage and SUVmax, though there was a significant difference between the BCR-free patients and BCT patients. In addition, the measurement of the maximal diameters on PET/CT is tricky though all measurements in the present study were performed by the same team with the same methods. Therefore, the value of maximal tumor diameters needs to be further verified and optimized in the following study.

In conclusion, a PSMA-ligand PET/CT-based risk model was developed for the BCR prediction following RP. Our newly developed risk model was shown to have better efficacy in predicting 2-year BCR after RP than the current D’Amico and CAPRA nomograms. Furthermore, the efficacy of the existing models were significantly improved by the additions of the parameters derived from PSMA-ligand PET/CT. PSMA-ligand PET/CT-based risk model showed great potential for the risk stratification and prediction of BCR of localized PCa after RP, which needed to be further validation by prospective studies.

## Conclusion

This study demonstrated that a PSMA-ligand PET/CT based model had a good efficacy in predicting 2-year BCR after RP and the efficacy of the existing models were significantly improved by the additions of the parameters derived from PSMA-ligand PET/CT.

## Data Availability Statement

The original contributions presented in the study are included in the article/supplementary material. Further inquiries can be directed to the corresponding authors.

## Ethics Statement

The studies involving human participants were reviewed and approved by 2017-147-01. The patients/participants provided their written informed consent to participate in this study. Written informed consent was obtained from the individual(s) for the publication of any potentially identifiable images or data included in this article.

## Author Contributions

All authors listed have made a substantial, direct, and intellectual contribution to the work and approved it for publication.

## Funding

This study was supported by grants from the National Natural Science Foundation of China (81602232, 81802535), Nanjing Medical Science and technique Development Foundation (QRX17128), and Nanjing Health Distinguished Youth Fund (JQX16025). All the funding supported equally in the design of the study and collection, analysis, and interpretation of data and in writing the manuscript.

## Conflict of Interest

The authors declare that the research was conducted in the absence of any commercial or financial relationships that could be construed as a potential conflict of interest.

## Publisher’s Note

All claims expressed in this article are solely those of the authors and do not necessarily represent those of their affiliated organizations, or those of the publisher, the editors and the reviewers. Any product that may be evaluated in this article, or claim that may be made by its manufacturer, is not guaranteed or endorsed by the publisher.
